# Discovery of an Internal α-1,3-Linkage-Containing Manno-Oligosaccharide in the *O*-Linked Cell Wall Mannan of Two *Candida parapsilosis* Complex Species via a Benanomicin A-Based Purification Method

**DOI:** 10.3390/microorganisms14071472

**Published:** 2026-07-04

**Authors:** Kei Higashijima, Takuya Kuraoka, Norihiko Fujimatsu, Akihiro Ambo, Fumie Ito, Yukiko Ogawa, Hidemitsu Kobayashi

**Affiliations:** 1 Laboratory of Microbiology, Department of Pharmacy, Faculty of Pharmaceutical Science, Nagasaki International University, 2825-7 Huis Ten Bosch, Sasebo 859-3298, Nagasaki, Japan; 2231d02@st.niu.ac.jp (K.H.); kuraoka@niu.ac.jp (T.K.); masarumeilpowerjunior@gmail.com (N.F.); 2 Center for Laboratory Animal Science, Faculty of Pharmaceutical Sciences, Tohoku Medical and Pharmaceutical University, 4-4-1 Komatsushima, Aoba-ku, Sendai 981-8558, Miyagi, Japan; aaambo@tohoku-mpu.ac.jp; 3 Division of Infection and Host Defense, Faculty of Pharmaceutical Sciences, Tohoku Medical and Pharmaceutical University, 4-4-1 Komatsushima, Aoba-ku, Sendai 981-8558, Miyagi, Japan; fumieito@tohoku-mpu.ac.jp; 4 Laboratory of Infection Control, Department of Pharmacy, Faculty of Pharmaceutical Science, Nagasaki International University, 2825-7 Huis Ten Bosch, Sasebo 859-3298, Nagasaki, Japan; yogawa@niu.ac.jp

**Keywords:** *Candida parapsilosis*, *Candida orthopsilosis*, *Candida metapsilosis*, Cell wall mannan, *O*-linked oligosaccharide, Benanomicin A, α-1,3-linkage, budding yeast

## Abstract

Cell wall mannan samples were prepared from a single strain of each *Candida parapsilosis* complex species (*C. parapsilosis* sensu stricto, *C. orthopsilosis*, and *C. metapsilosis*) using benanomicin A, an antibiotic with lectin-like activity. Their *O*-linked oligosaccharides were subsequently obtained in good yield via β-elimination (alkaline treatment). From the mannan of *C. parapsilosis sensu stricto*, only short α-1,2-linked manno-oligosaccharides were obtained. In contrast, the mannans of *C. orthopsilosis* and *C. metapsilosis* additionally yielded a unique pentasaccharide, Manα1-2Manα1-3Manα1-2Manα1-2Man. Notably, this pentasaccharide, which contains an internal α-1,3-linked mannose residue, was recovered in appreciable amounts, whereas the corresponding tetrasaccharide (Manα1-3Manα1-2Manα1-2Man), a presumed biosynthetic intermediate, was barely detectable. This structure represents, to our knowledge, the first example of such a manno-oligosaccharide isolated from the *O*-linked domain of budding yeast cell wall mannans.

## 1. Introduction

*Candida parapsilosis* has long been recognized as an important opportunistic yeast pathogen, particularly in neonates and immunocompromised patients. However, molecular typing studies revealed that clinical isolates previously identified as *C. parapsilosis* complex (CPC) actually comprise three genetically distinct lineages. On the basis of multilocus sequence data and DNA fingerprinting, these lineages were formally elevated to species rank in 2005 and are now recognized as *Candida parapsilosis* sensu stricto (hereafter referred to as *C. parapsilosis*), *Candida orthopsilosis*, and *Candida metapsilosis* [1,2].

Members of the CPC have emerged as important causes of bloodstream and device-related infections, particularly in neonates and other vulnerable patients [3,4,5], and they may differ in virulence-related traits such as adhesion, biofilm formation, and the production of hydrolytic enzymes [5,6,7,8]. In addition, species-specific patterns of antifungal susceptibility and the emergence of resistant isolates have been reported, underscoring the clinical relevance of distinguishing among them [5,9,10]. Because the three species of the CPC are phenotypically very similar and cannot be reliably distinguished by routine morphological or biochemical tests, molecular methods are usually required for accurate identification [1,3,11,12].

As a rapid method for distinguishing among the three CPC species, an immunochemical approach to detect species-specific target antigens can be considered. In budding yeasts, most of the antigenic activity is attributable to mannan molecules located in the outermost cell wall layer. In *Candida* species, these mannans consist of *N*-linked manno-polysaccharides attached to asparagine and *O*-linked manno-oligosaccharides attached to serine or threonine, as demonstrated by structural and immunochemical studies of *Candida* mannans [13]. There is also clear evidence that both *O*- and *N*-linked mannosylation are crucial for the cell wall architecture and innate immune recognition of the pathogenic genus *Candida* [14,15]. Despite the significant clinical importance of distinguishing the three CPC species, comparative information on the detailed structures of the cell wall mannans responsible for their antigenicity remains limited. The *N-*linked mannan of *C. parapsilosis* has already been characterized in detail by Shibata and colleagues [16]. On the other hand, *O*-linked glycans have not been investigated in any of the three CPC species; thus, the present study focuses on elucidating their *O*-linked glycan structures.

Accurate structural analysis of cell wall mannans, especially of labile *O*-linked oligosaccharide chains, requires preparation methods that selectively isolate mannan while preserving the native structures of its side chains. Benanomicin A, a mannan-binding antibiotic, is a useful tool for obtaining cell wall mannan fractions with intact *O*-linked side chains, providing a basis for comparative structural studies of *O*-linked oligosaccharides [17]. In this study, we utilized this benanomicin A-based mannan preparation method to investigate the structural differences in cell wall mannans among three strains: *C. parapsilosis* NBRC 0708, *C. orthopsilosis* NBRC 0585, and *C. metapsilosis* NBRC 0640, with a particular emphasis on their *O*-linked oligosaccharides.

## 2. Materials and Methods

### 2.1. General

*C*. *parapsilosis* NBRC 0708, *C*. *orthopsilosis* NBRC 0585, and *C. metapsilosis* NBRC 0640 were obtained from the Biological Resource Center (NBRC), National Institute of Technology and Evaluation (NITE), Japan. Cells were cultivated in yeast extract–Sabouraud liquid medium [0.5% (*w*/*v*) yeast extract, 1% (*w*/*v*) peptone, and 2% (*w*/*v*) glucose] at 27 °C for 72 h on a reciprocal shaker. Benanomicin A was kindly provided by Dr. Shuichi Gomi (Pharmaceutical Research Center, Meiji Seika Kaisha, Ltd., Tokyo, Japan).

### 2.2. Purification of Cell Wall Mannans of C. parapsilosis, C. orthopsilosis, and C. metapsilosis Using Benanomicin A

Crude extracts were obtained from dried cells of *C. parapsilosis*, *C. orthopsilosis*, and *C. metapsilosis* as described previously [18]. Each mannan fraction was prepared using benanomicin A according to the method of Kuraoka et al. [17]. Briefly, 500 mg of the crude extract was dissolved in water, and 25 mL of 0.2% (*w*/*v*) benanomicin A in 0.2 M CaCl_2_ was added under vigorous stirring. After 2 h, the resulting red precipitate (benanomicin A–mannan complex) was collected by centrifugation at 1450× *g* for 10 min. The pellet was rinsed with 25 mL of 0.2 M CaCl_2_ under vigorous stirring and transferred to a 100-mL beaker. Next, a mixture containing 20 mL of 0.2 M EDTA⋅2Na and 20 mL of 0.01 M HCl was added to the precipitate. After 10 min, the resulting precipitate (comprising benanomicin A and the EDTA-Ca⋅2Na chelate) was removed by centrifugation at 1450× *g* for 10 min. The supernatant was neutralized with 0.1 M NaOH, dialyzed against running tap water for 48 h, concentrated in vacuo to 5 mL, and lyophilized. The mannans purified by this procedure are referred to as fractions Par, Ort, and Met, respectively.

### 2.3. Alkaline Treatment (β-Elimination) of Fractions Par, Ort, and Met

Alkaline β-elimination was performed as described previously [19]. Fractions Par, Ort, and Met were each dissolved in 30 mL of 0.1 M NaOH, and the solutions were incubated at 25 °C for 18 h. The mixture was then neutralized with 1 M HCl, concentrated to a small volume, and applied to a Bio-Gel P-2 column (2.5 × 100 cm; Bio-Rad, Tokyo, Japan). Elution was performed with water at a flow rate of 0.25 mL/min. The residual polysaccharides remaining after this process, corresponding to the *N*-linked mannans, are referred to as fractions Par-b, Ort-b, and Met-b, respectively.

### 2.4. Instrumental Analysis

^1^H-nuclear magnetic resonance (^1^H-NMR) spectra were recorded by means of a JEOL ECZ500R spectrometer (JEOL Ltd., Tokyo, Japan), in accordance with previous description [20]. Each oligosaccharide sample (4 mg each) was dissolved in D_2_O solution at 45 °C, using acetone as the standard (2.217 ppm).

Matrix-Assisted Laser Desorption/Ionization Time-of-Flight Mass Spectrometry (MALDI-TOF MS) was performed under the following conditions. Pentasaccharides were analyzed by MALDI-TOF MS (Autoflex II, Bruker Daltonics, Billerica, MA, USA) using standard protocols in reflector positive ion mode. 2,5-dihydroxybenzoic acid (2,5-DHB) was used as the matrix. 2,5-DHB was dissolved in 50% acetonitrile (*v*/*v*) solution at a concentration of 1 mg/mL. Samples were dissolved in ultrapure water and adjusted to 0.25 mg/mL, 0.5 mg/mL, and 1 mg/mL. Equal volumes (3 μL each) of sample and DHB solutions were mixed thoroughly, and 1 μL of the mixture was spotted onto a MALDI plate, air-dried, and submitted to analysis. Laminaripentasaccharide was used for calibration.

### 2.5. Quantification and Calculation of O-Linked Oligosaccharide Molar Ratios

The relative distribution of *O*-linked oligosaccharides was determined based on the peak areas obtained from the gel filtration elution profiles. The relative molar amount of each oligosaccharide was calculated by dividing its peak area by the corresponding molecular weight. To compare the oligosaccharide profiles among the three CPC species, the molar ratio of each oligosaccharide was expressed relative to that of the disaccharide from fraction Par, which was defined as 1.0. The molar ratio was calculated using the following equation: molar ratio = (*A_i_*/*MW_i_*)/(*A_di-Par_*/*MW_di-Par_*), where *A_i_* and *MW_i_* represent the peak area and molecular weight of a given oligosaccharide *i*, respectively, and *A_di-Par_* and *MW_di-Par_* represent the peak area and molecular weight of the disaccharide obtained from fraction Par, respectively.

### 2.6. Analytical Methods

Total carbohydrate content was determined by the phenol–H_2_SO_4_ method of Dubois et al. [21] using D-mannose as a standard. Total protein content was determined by the Folin method [22] using bovine serum albumin as a standard. Total phosphate content was determined by the method of Ames–Dubin [23] using KH_2_PO_4_ as the standard.

## 3. Results

### 3.1. Chemical Composition of Mannan Fractions of CPC Species

Table 1 shows the results of chemical analyses of the mannan fractions Par, Ort, and Met prepared with benanomicin A, as well as the corresponding mannan fractions Par-b, Ort-b, and Met-b that lost their *O*-linked sugar chains due to alkaline treatment. Fractions Par, Ort, and Met were composed mostly of carbohydrates, containing small amounts of protein and only trace amounts of phosphate. Given that low-molecular-weight impurities were thoroughly removed by the final dialysis against running water, this trace level of phosphate indicates indicate that the isolated materials are effectively free from significant phosphorus-containing contaminants, such as nucleic acids, ensuring that there is no interference with subsequent structural analyses. Furthermore, the remaining portion of the fractions is most likely attributed to the hygroscopic nature of the purified mannoprotein complex and associated moisture. In fractions Par-b, Ort-b, and Met-b, the relative protein contents increased as a result of the β-elimination of *O*-linked glycans. The increase in protein content was more evident in fractions Ort-b and Met-b than in fraction Par-b, suggesting that relatively larger amounts of *O*-linked glycans were lost from their parental mannans (fractions Ort and Met).

### 3.2. Analysis of Mannan Fractions of CPC Species by ^1^H-NMR

**Figure 1:**
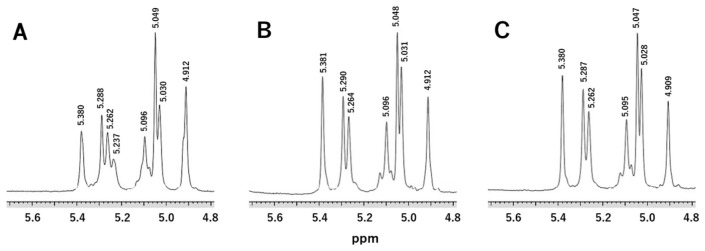
^1^H-NMR spectra (H-1 region) of mannans, fractions Par (**A**), Ort (**B**), and Met (**C**), obtained from three CPC species, *C. parapsilosis*, *C. orthopsilosis*, and *C. metapsilosis*, by Benanomicin A.

**Figure 2:**
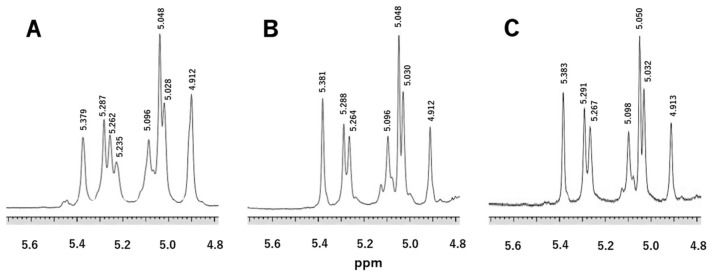
^1^H-NMR spectra (H-1 region) of alkaline-treated mannans, fractions Par-b (**A**), Ort-b (**B**), and Met-b (**C**), obtained from mannans, fractions Par, Ort, and Met.

To obtain information about the sugar chain structures of these mannans, ^1^H-NMR spectra were recorded for fractions Par, Ort, Met, Par-b, Ort-b, and Met-b (Figure 1 and Figure 2). The H-1 signal patterns of the three mannan fractions Par, Ort, and Met (Figure 1) reflect both *N*-linked and *O*-linked sugar chain structures and are quite similar to each other. However, a signal between 5.23 and 5.24 ppm was observed only in fraction Par and was not detected in the other mannan fractions, Ort and Met. Based on the work of Shibata et al. [16], this signal is attributed to the glycan structure Manα1-3(Manα1-6)Manα1-2Man, in which α-1,3-linked and α-1,6-linked mannose residues branch from an α-1,2-linked mannose residue. Shibata and co-workers previously demonstrated this assignment through detailed structural analysis of the *N*-linked mannan from *Candida albicans* [16]. The ppm values of the other common signals indicate that these mannans consist only of α-1,2-, α-1,3-, and α-1,6-linkages, containing no β-1,2-linkages or mannosyl-6-phosphate, which contribute to the presence of mannose phosphodiester bonds in the *N*-mannans of *C. albicans* and *Candida tropicalis* [16,24]. Furthermore, the H-1 signals of the mannan fractions Par-b, Ort-b, and Met-b shown in Figure 2 reflect only their *N*-linked sugar chain structures, as the *O*-linked sugar chains were removed by alkaline treatment. These signal patterns were very similar to those of fractions Par, Ort, and Met before the alkaline treatment. Although the intensities of some signals differed before and after the alkaline treatment, all signals were retained, suggesting that the *N*- and *O*-linked sugar chains may share similar glycan structures.

### 3.3. Fractionation of Alkaline-Labile Oligosaccharides by Gel Filtration

**Figure 3:**
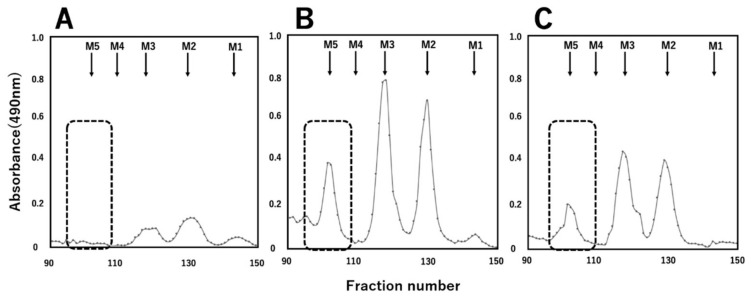
Elution profile of released oligosaccharides obtained from fraction Par (**A**), fraction Ort (**B**), and fraction Met (**C**) by alkaline treatment (β-elimination). Arrows indicate eluted positions of standard α-1,2-linked manno-oligosaccharides obtained from the *C. tropicalis* mannan [24]. M1, M2, M3, M4, and M5 indicate monosaccharaide, disaccharide, trisaccharide, tetrasaccharide, and pentasaccharide, respectively.

We next analyzed the structures of the *O*-linked sugar chains in more detail. Figure 3 shows the elution profiles obtained by fractionating the oligosaccharides released by alkaline treatment (β-elimination) on a Bio-Gel P-2 column. The yields of these oligosaccharides, expressed as weight percentages relative to the original mannans, were 3.2% for fraction Par, 20.8% for fraction Ort, and 12.1% for fraction Met. From fraction Par, only small amounts of short oligosaccharides, specifically a disaccharide and a trisaccharide, were recovered. In contrast, fractions Ort and Met yielded these short oligosaccharides along with a pentasaccharide in relatively high amounts (indicated by the dotted box), whereas a tetrasaccharide fraction was scarcely detected in any of the species. These oligosaccharide yields correlate well with the increases in protein content observed after the alkaline treatment in Table 1, indicating that the mannans of fractions Ort and Met contain a higher proportion of alkali-labile O-linked sugar chains than those of fraction Par.

### 3.4. Analysis of Alkaline-Labile Oligosaccharides by ^1^H-NMR and MALDI-TOF MS

**Figure 4:**
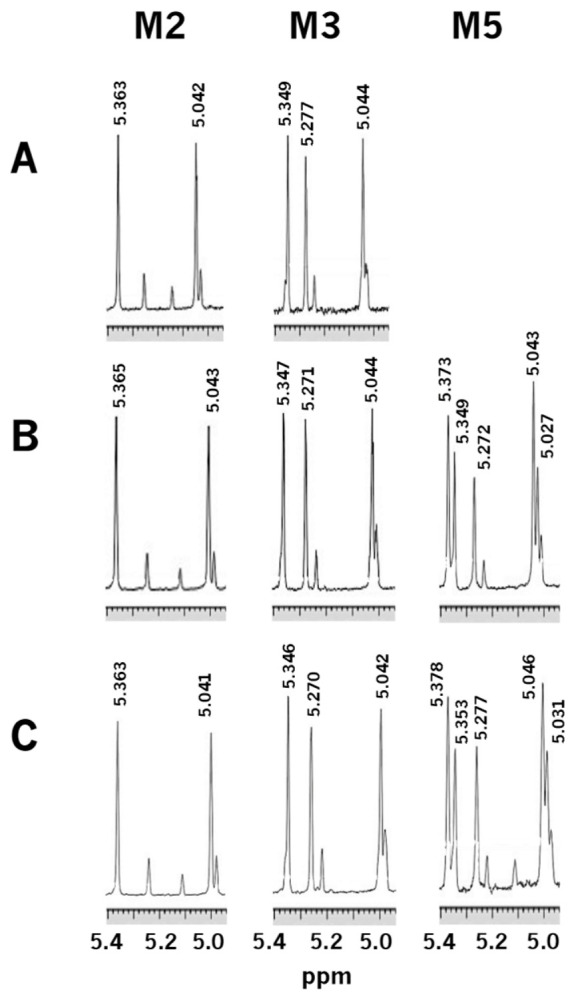
^1^H-NMR spectra (H-1 region) of released oligosaccharides obtained from three mannans of CPC species, fraction Par (**A**), fraction Ort (**B**), and fraction Met (**C**) by alkaline treatment (β-elimination). M2, M3, and M5 indicate disaccharide, trisaccharide, and pentasaccharides, respectively.

The results of the ^1^H-NMR analyses of the oligosaccharides generated by the alkaline treatment of the three CPC mannan fractions Par, Ort, and Met are shown in Figure 4. The chemical shift values of the H-1 signals indicated that all disaccharides and trisaccharides obtained from the three mannans consisted solely of α-1,2-linked mannose residues (Figure 4A–C). In contrast, the pentasaccharides obtained selectively from fractions Ort and Met exhibited two characteristic signals at 5.374 and 5.037 ppm (Figure 4B,C). These H-1 signals corresponded to mannose residues with α1-2Manα1-3 and α1-3Manα1-2 linkages, respectively, indicating that the chemical structure of these two pentasaccharides was Manα1-2Manα1-3Manα1-2Manα1-2Man. Crucially, the ^1^H-NMR signal pattern of this pentasaccharide was identical to that of the pentasaccharide obtained via acetolysis of the cell wall *N*-linked mannan from *Saccharomyces kluyveri*, as previously reported by Shibata et al. [25]. The chemical shift values of the H-1 signals corresponding to each mannose residue of the oligosaccharides obtained from fraction Ort are summarized in Table 2. Furthermore, MS analysis of these oligosaccharides showed that the observed m/z values of the pentasaccharides derived from fractions Ort and Met were 851.444 [M+Na]^+^ and 851.329 [M+Na]^+^, respectively, which supported the interpretation of the ^1^H-NMR data obtained for the oligosaccharides produced by the alkaline treatment.

### 3.5. Distribution of O-Linked Oligosaccharides in the Cell Wall Mannans of CPC Species

**Figure 5:**
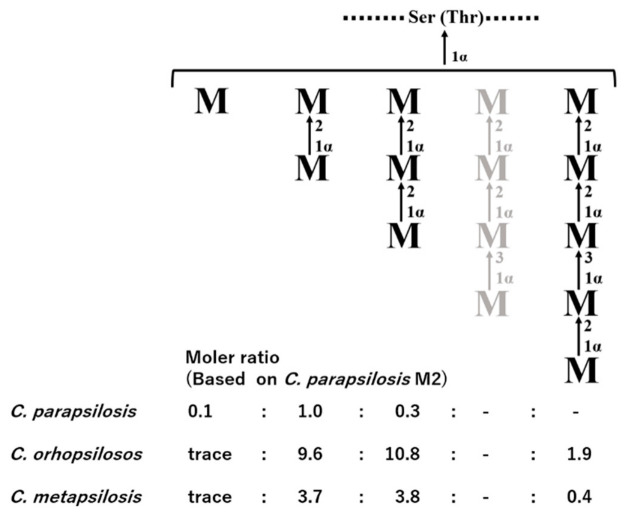
Distribution of *O*-linked oligomannosyl chains in cell wall mannans obtained from three CPC species, *C. parapsilosis*, *C. orthopsilosis*, and *C. metapsilosis*. M, Ser, and Thr denote D-mannose, L-serine, and L-threonine, respectively. The chain sequence is not specified. The molar ratios of *O*-linked oligosaccharide chains in the mannans from the three CPC species were calculated from the areas under the elution curves in Figure 3, using the disaccharide obtained from *C. parapsilosis* as a reference value (set to 1.00).

Figure 5 summarizes the distribution of *O*-linked oligosaccharides attached to Ser/Thr residues in the three CPC mannans. The molar amount of each *O*-linked oligosaccharide is expressed as ratios relative to the disaccharide, Manα1-2Man, in fraction Par, which was set to 1. Although minimal differences were observed in the protein content of the mannan molecules from the three CPC species (Table 1), the distribution of their *O*-linked oligosaccharide chains differed remarkably. Specifically, the proteins in the mannan of *C. parapsilosis* carry only a very small number of *O*-linked sugar chains. In contrast, those in the mannans of the other two species, *C. orthopsilosis* and *C. metapsilosis*, are substituted with significantly more *O*-linked sugar chains, including the unique pentasaccharide that is not found in *C. parapsilosis*.

## 4. Discussion

The typical *O*-linked oligosaccharides of budding yeast such as *Saccharomyces cerevisiae* consist of short α-1,2-linked mannose chains optionally capped with a terminal α-1,3-linked mannose residue [26,27]. In commonly studied *Candida* species, *O*-linked glycans, which are typically present in only small amounts within the cell wall mannan, have generally been described as short α-1,2-linked oligosaccharides lacking α-1,3-linked mannose residues [24,28].

In this study, we successfully isolated *O*-linked glycans utilizing benanomicin A, an antifungal antibiotic with mannose-specific lectin-like activity, via our previously developed cell wall mannan preparation method [17]. This approach enabled the discovery of a unique *O*-linked oligosaccharide chain (Manα1-2Manα1-3Manα1-2Manα1-2Man) in the cell wall mannans of *C. orthopsilosis* and *C. metapsilosis*. To our knowledge, this structure represents the first reported example of an *O*-linked oligosaccharide derived from a budding yeast that contains an internal α-1,3-linked mannose residue.

Moreover, the present results revealed marked interspecies differences in *O*-linked oligosaccharides architecture within the CPC. Under our experimental conditions, *C. parapsilosis* displayed only short α-1,2-linked *O*-linked chains composed of three mannose residues or fewer, whereas *C. orthopsilosis* and *C. metapsilosis* possessed longer *O*-linked chains of up to five mannose residues that contained an internal α-1,3-linked mannose within a linear backbone. Notably, the corresponding putative biosynthetic intermediate, a tetrasaccharide with a non-reducing terminal α-1,3-linked mannose, Manα1-3Manα1-2Manα1-2Man, was only rarely detected, leading us to speculate that this intermediate is either very transient or is rapidly elongated to the observed pentasaccharide. One plausible hypothesis for these distinct “end-point” structures is that *C. parapsilosis* (sensu stricto) lacks a functional α-1,3-mannosyltransferase for *O*-oligosaccharides biosynthesis, or expresses it at very low levels, whereas *C. orthopsilosis* and *C. metapsilosis* retain an active α-1,3-mannosyltransferase that introduces the internal α-1,3 linkage followed by efficient α-1,2-mediated elongation. In this scenario, although further studies using a larger number of clinical isolates and genetic analyses are required to confirm this, species-specific differences in the presence or activity of the relevant α-1,3-mannosyltransferase are proposed as a potential determinant of whether short, purely α-1,2-linked *O*-oligosaccharides or longer chains containing an internal α-1,3 linkage accumulate as the predominant *O*-linked structures.

The chemical structure of the *N*-linked site of the *C. parapsilosis* cell wall mannan has already been reported by Shibata et al. [16]. In that study, a hexasaccharide, Manα1-2Manα1-3Manα1-2Manα1-2Manα1-2Man, containing an internal α-1,3 linkage, was reported to be present as a side chain of the *N*-linked mannan in *C. parapsilosis*. This hexasaccharide is structurally similar on the non-reducing terminal side to the *O*-linked pentasaccharide, Manα1-2Manα1-3Manα1-2Manα1-2Manα1-Ser/Thr, that we identified in the mannans of *C. orthopsilosis* and *C. metapsilosis* in the present study. Importantly, monoclonal antibodies against glycosphingolipids have been shown to distinguish glycoepitopes depending on the nature of the aglycone at the reducing end, such as different ceramide structures [29,30]. Therefore, if these *O*-linked oligosaccharides are indeed exposed on the outer surface of the cell wall, as suggested by the cell wall architecture model of Gow et al. [31], the structural differences between these *N*- and *O*-linked glycans could potentially be exploited as target antigens and the rapid identification of *C. parapsilosis* and its discrimination from the other two species is expected to be feasible.

However, several limitations of this study should be noted. Because our data and interspecies comparisons are based on a single preparation of each mannan, the present findings do not account for potential batch-to-batch or strain-to-strain variability in oligosaccharide yields. Therefore, direct quantitative comparisons of yields among these species must be interpreted with caution. Further investigations involving multiple independent preparations and advanced structural validation, such as two-dimensional NMR analysis, will be valuable to firmly confirm the consistency and variability of these oligosaccharide yields.

We have also nearly completed the structural analysis of *N*-linked mannans in *C. orthopsilosis* and *C. metapsilosis* and plan to report these findings in the near future. Together with the present characterization of *O*-linked mannan chains, these data will provide a more comprehensive picture of the mannan structures that constitute the outermost layer of the *Candida* cell wall. Elucidating the detailed chemical structures of these mannans is of fundamental importance for biological and molecular studies of yeast mannan biosynthesis [32,33]. Furthermore, such structural insights are essential for a deeper understanding of how pathogenic yeasts interact with their hosts [34,35], including their recognition by immune receptors and their ability to modulate host immune responses and express virulence traits [36,37]. In this context, the present work represents an important step towards linking species-specific mannan architectures in the CPC to their underlying biosynthetic pathways and to their potential roles in host–pathogen interactions. While our work represents an important step, future comparative studies utilizing standardized, multiple preparations will be necessary to fully generalize these species-specific variations in mannan architecture and their biological implications.

## Figures and Tables

**Table 1:** Chemical composition of mannan fractions, Par, Ort, Met, Par-b, Ort-b, and Met-b, obtained from CPC species.

Mannan Fraction	Total Carbohydrate (%) ^a^	Total Protein (%) ^b^	Total Phosphate (%) ^c^	Yield (%) ^d^
Par	80.43	3.0	trace	-
Ort	71.83	4.4	trace	-
Met	70.02	4.0	trace	-
Par-b	80.18	4.4	trace	79.6
Ort-b	73.50	14.4	trace	61.2
Met-b	76.84	12.4	trace	60.7

^a^ Determined by the phenol/sulfuric acid method [21] as D-mannose. ^b^ Determined by the Lowry–Folin method of Lowry et al. [22] with bovine serum albumin as the standard. ^c^ Determined by Ames–Dubin method [23] as -H_2_PO_4_. ^d^ Weight-based fractions Par, Ort, and Met, respectively.

**Table 2:** Assignment of H-1 chemical shifts for manno-oligosaccharides obtained from fraction Ort produced by alkaline treatment (β-elimination).

	Oligosaccharide					H-1 Chemical Shift				
	*E*	*D*	*C*	*B*	*A*	*E*	*D*	*C*	*B*	*A*
M2				Manα1→	2Man				5.043	5.365
M3			Manα1→	2Manα1→	2Man			5.044	5.271	5.347
M5	Manα1→	2Manα1→	3Manα1→	2Manα1→	2Man	5.043	5.373	5.027	5.272	5.349

M2, M3, and M5 refer to disaccharides, trisaccharide, and pentasaccharide, respectively. *A* designates reducing terminal mannose residue. *B*, *C*, and *D* designate internal mannose residues. *E* designates non-reducing terminal mannose residue.

## Data Availability

The raw data supporting the conclusions of this article will be made available by the authors on request.

## Abbreviations

CPC: *Candida parapsilosis* complex, ^1^H-NMR: proton nuclear magnetic resonance, MALDI-TOF MS: Matrix-Assisted Laser Desorption/Ionization Time-of-Flight Mass Spectrometry.
